# Proteins and Carbohydrates from Red Seaweeds: Evidence for Beneficial Effects on Gut Function and Microbiota

**DOI:** 10.3390/md13085358

**Published:** 2015-08-20

**Authors:** Raúl E. Cian, Silvina R. Drago, Fermín Sánchez de Medina, Olga Martínez-Augustin

**Affiliations:** 1 Instituto de Tecnología de Alimentos, Facultad de Ingeniería Química, Universidad Nacional del Litoral, 1° de Mayo 3250, (3000) Santa Fe, República Argentina; E-Mails: rec_704@yahoo.com.ar (R.E.C.); sdrago@fiq.unl.edu.ar (S.R.D.); 2 Consejo Nacional de Investigaciones Científicas y Técnicas (CONICET), Av. Rivadavia 1917 (C1033AAJ), Ciudad Autónoma de Buenos Aires, República Argentina; 3 Department of Pharmacology, Centro de Investigación Biomédica en Red de Enfermedades Hepáticas y Digestivas (CIBERehd), Universidad de Granada, Campus de Cartuja s/n, 18071 Granada, Spain; E-Mail: fsanchez@ugr.es; 4 Instituto de Ciencia y Tecnología de los Alimentos José Mataix, Universidad de Granada, 18071 Granada, Spain; 5 Department of Biochemistry and Molecular Biology II, Centro de Investigación Biomédica en Red de Enfermedades Hepáticas y Digestivas (CIBERehd), Universidad de Granada, Campus de Cartuja s/n, 18071 Granada, Spain; 6 Instituto de Investigación Biosanitaria. ibs. GRANADA, University of Granada, 18071 Granada, Spain

**Keywords:** red seaweeds, sulfated galactans, bioactive peptides, Rhodophyta, mucosal barrier function, immunomodulation, cell differentiation, cell proliferation, NF-κB, MAPK

## Abstract

Based on their composition, marine algae, and namely red seaweeds, are good potential functional foods. Intestinal mucosal barrier function refers to the capacity of the intestine to provide adequate containment of luminal microorganisms and molecules. Here, we will first outline the component of seaweeds and will summarize the effects of these on the regulation of mucosal barrier function. Special attention will be paid to unique components of red seaweeds: proteins and derived peptides (e.g., phycobiliproteins, glycoproteins that contain “cellulose binding domains”, phycolectins and the related mycosporine-like amino acids) together with polysaccharides (e.g., floridean starch and sulfated galactans, such as carrageenans, agarans and “dl-hybrid”) and minerals. These compounds have been shown to exert prebiotic effects, to regulate intestinal epithelial cell, macrophage and lymphocyte proliferation and differentiation and to modulate the immune response. Molecular mechanisms of action of peptides and polysaccharides are starting to be elucidated, and evidence indicating the involvement of epidermal growth factor receptor (EGFR), insulin-like growth factor receptor (IGFR), Toll-like receptors (TLR) and signal transduction pathways mediated by protein kinase B (PKB or AKT), nuclear factor-κB (NF-κB) and mitogen activated protein kinases (MAPK) will also be summarized. The need for further research is clear, but *in vivo* experiments point to an overall antiinflammatory effect of these algae, indicating that they can reinforce membrane barrier function.

## 1. Introduction

Seaweeds represent a considerable part of the ocean biomass (mainly located on the coastline) and their use as food dates back to 2700 BC in China [1]. This practice remains widespread currently in Eastern countries such as China, Japan, Korea, *etc.* [2]. However, in Western countries, direct human consumption is unusual due to cultural reasons and consumer habits. In this regard, the main use in these countries is as source of hydrocolloids and their subsequent application as thickeners and gelling agents in the food industry [3]. The potential production of bioactive compounds is another use, introduced in the market by the pharmaceutical and/or cosmetic industry [4].

Recently, it has been shown that the consumption of seaweed in Asian countries is associated with a low incidence of cancers compared to European and North American countries [5]. Furthermore, other putative beneficial health effects have been identified, such as decreased blood pressure and blood sugar, anti-inflammatory, immunomodulatory and neuroprotective effects, among others. A mechanistic link has been proposed due to the presence in algae of different bioactive compounds, including sulfated polysaccharides, polyphenols, carotenoids, amino acids, proteins/peptides and lipids [6]. Because of their substantial diversity and composition, red seaweeds (*i.e.*, Rhodophyta) have aroused significant interest in the food and the pharmaceutical industry for the search of new natural nutrients and bioactive compounds. It is noteworthy that, among seaweeds, red algae contain high amounts of carbohydrates, proteins and minerals [7]. Specific functional properties have been attributed to Rhodophyta proteins/peptides and polysaccharides because of their unique composition. Indeed, these polysaccharides have chemical structures and physicochemical properties that differ substantially from those of land plants [8].

Recently, the concept of intestinal mucosal barrier function (MBF) has gained much attention. Intestinal MBF is the capacity of the intestine to provide adequate containment of luminal microorganisms and molecules while preserving the ability to absorb nutrients. The link between alterations in the intestinal MBF and intestinal diseases has been known for a while. For instance, increased intestinal permeability or dysbiosis (alterations in the composition of the intestinal microbiota, itself a component of the mucosal barrier) have been related to irritable bowel syndrome (IBS) or inflammatory bowel disease [9,10]. Interestingly, recent advances have also shown that systemic diseases, like metabolic syndrome, allergy, chronic kidney disease or hepatic inflammation, can be related to modifications in intestinal MBF [11,12,13,14]. Therefore, MBF could be an important target to prevent or treat these diseases. Based on their prebiotic, antioxidant or immunomodulatory properties, red seaweeds and their components may be considered as functional foods that can modify MBF components in an advantageous way. Here, we discuss red seaweed composition and summarize recent advances in the study of functional properties of red seaweeds, focusing on those related to modulation of intestinal MBF. Special attention is paid to red seaweed proteins and polysaccharides because of their specific structure and composition.

## 2. Red Seaweed Composition

Red seaweeds or Rhodophyta (from the Greek rhodo- “rose” and -phyta “plant”), are phylogenetically very old organisms, having many peculiarities in morphology and mode of reproduction [15]. They are classified as non-vascular plants from the *Primoplantae* clade and belong to a group of around 6100 species with a wide variety of shapes and sizes. Rhodophyta are photosynthetic, lack flagella and contain chlorophyll a and d, as well as accessory pigments such as carotenoids and phycobiliproteins (phycoerythrin, phycocyanin, and allophycocyanin) [16]. As noted, red seaweeds have a unique polysaccharide composition and do not have starch in chloroplasts, using floridean starch from cytoplasm as reserve [17].

Although red seaweeds are found in all latitudes, there is a marked abundance in equatorial regions. There are few species in polar and sub-polar regions, where brown and green algae predominate. Larger species of red algae with massive thalli appear in cold and temperate areas, while in tropical seas red algae are mainly small filamentous plants. Rhodophytas have greater ability to live at great depths than other algae groups and they can grow at up to 200 m deep, a skill related to the presence of accessory pigments [18].

## 3. Red Algae Cell Wall Components

The cell wall of red seaweeds accounts for up to 65% (*w*/*w*) of dry matter and comprises three domains: fibrillar wall, amorphous matrix and glycoprotein domain. The fibrillar polysaccharides and glycoprotein domains form a reticulated cell wall which is embedded in the amorphous matrix. Fibrillar polysaccharides are the most inert and resistant cell wall component, with cellulose being the most important element. The backbone of cellulose is d-glucose units linked by β-(1→4) bonds. In some cases cellulose may be substituted by polymers containing β-d-mannose or β-d-xylose units linked by β-(1→4) or β-(1→3) bonds, respectively [19]. The glycoprotein domain is little known, but it is constituted by glycoproteins that contain “cellulose binding domains” which promote crosslinking of polysaccharide fibers. Finally, the amorphous matrix consists of sulfated galactans, polysaccharides that contain multiple units of the monosaccharide galactose with sulfate ester, such as carrageenans, agarans and “dl-hybrid” [20], and usually extends to intercellular spaces between adjacent cells. These polysaccharides are named phycocolloids for their ability to form aqueous gels [21]. The sulfated galactans that form the amorphous matrix have a structure based on the repetition of two different subunits, A and B. The A unit is formed by β-galactose residues with d-conformation, while the B unit consist of α-galactose residues with D and L conformation in carrageenans and agarans, respectively. Both form a linear backbone of alternating 3-linked β-d-galactopyranose and 4-linked α-galactopyranose residues. In some cases, the B unit may exist in the form of 3,6-anhydrogalactopyranose. Various hydroxy groups may be substituted by ester sulfate, methyl groups, pyruvic acid acetal and sometimes by additional monosaccharides [20,22].

**Figure 1:**
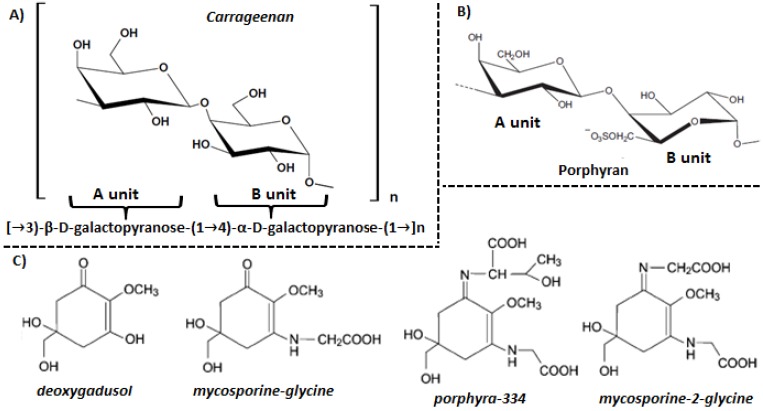
Carragenan (**A**), porphyran (**B**) and mycosporine-like amino acid structure.

Carrageenans are mainly synthesized by read algae of the Gigartinales order (*Gigartin*a, *Chondrus crispus*, *Eucheuma* and *Hypnea*), although they also appear in other Rhodophyta species [23]. They have a β-d-galactopyranose repetitive basic structure (A unit) and an α-d-galactopyranose (B unit) (Figure 1A). The degree of sulfation is higher than that of agarans [20]. The carrageenans structure is defined by the number and position of sulfate groups, the presence of 3,6-anhydro-d-galactose and the pyranosidic ring conformation [24]. There are about 15 prototypic carrageenan structures traditionally identified by Greek letters, of which the ones with the largest commercial interest are kappa (κ)-, iota (ι)- and lambda (λ)-carrageenan [20]. Their differences in chemical composition and configuration are responsible for their interesting rheological properties which make them useful as gelling, stabilizing and thickening agents in the food, pharmaceutical and cosmetics industry [21]. It is noteworthy that the red seaweed carrageenans may be chemically modified with one or more substitutions in the same molecule in different ratios, resulting in new types of carrageenans called “hybrid carrageenans”.

All carrageenans are water soluble, and insoluble in organic solvents, oil or fats. Water solubility is modulated to a great extent by the amount of sulfate groups present in the molecule, which augment hydrophilicity, and by their associated cations. The main cations found in carrageenans are sodium, potassium, calcium and magnesium, but other ions can also occur at lower frequency. The proportion of sulfate fractions and the equilibrium of cations in the water solution determine the viscosity of solutions or strength of gels formed by carrageenans [23]. Therefore, these are important factors for food and pharmaceutical industries that, as stated above, use carrageenans as thickening, gelling, and stabilizing agents.

Agarans are mainly synthesized by red seaweeds belonging to the *Pyropia*, *Gelidium*, *Gracilaria* and *Pterocladia* genera [3]. Agarans are composed mainly of the alternating 3-linked-β-d-galactopyranose unit (A unit) and the 4-linked-3,6-anhydro-α-l-galactopyranose unit (B unit) [3]. Structural variability is provided by the presence of substituent groups such as sulfate, methoxy and pyruvic [25]. Agarans synthesized by the *Pyropia*, *Porphyra* and *Bangia* genera are generically called porphyrans [26]. Their basic structure is similar to that of agarose, where the A unit can be formed by β-d-galactose and 6-O-methyl-β-d-galactose, while the B unit may be α-l-galactose, α-l-galactose-6-sulfate or 3,6-anhydro-α-l-galactose (Figure 1B) [27]. Thus porphyrans are characterized by a high substitution pattern in the A unit and at least a 50% B unit substitution [28]. These polysaccharides comprise the hot water soluble portion of the cell wall and are the main components of marine red algae [27].

## 4. Cytoplasm and Chloroplasts Components

### 4.1. Mycosporine-Like Amino Acids

Mycosporine-like amino acids (MAAs) are secondary metabolites of low molecular weight (<400 Da) which are composed by a cyclohexenone or cyclohexenimine chromophore conjugated with the nitrogen substituent of an amino acid or its imino alcohol. MAAs have a high denaturation temperature and are water soluble due to their ampholyte nature, and display absorption maxima ranging from 310 to 362 nm [29,30]. Generally, the ring system contains a glycine subunit linked to the third carbon atom. Some MAAs also contain sulfate esters or glycosidic linkages through the imine substituents. Differences between the absorption spectra of MAAs are due to the attached side groups and nitrogen substituents. They also have high photostability and low fluorescence emission [31].

Up to 23 MAAs from different marine organisms have been described, such as deoxygadusol, asterina-330, mycosporine-glycine, porphyra-334, mycosporine-2-glycine, *etc.* (Figure 1C) [32]. The biosynthesis has been predicted to occur via the first part of the *shikimate pathway*, but conclusive evidence is lacking. It has been found that 3-dehydroquinate, which is formed in the central steps of the *shikimate pathway*, acts as a precursor for the synthesis of fungal mycosporines and MAAs via gadusols. The primary MAA mycosporine-glycine, synthesized in the *shikimate pathway*, is then transformed into other secondary MAAs [33]. It is important to note that red seaweeds biosynthesize MAAs while other marine organisms acquire MAAs through the diet or by symbiotic or bacterial associations [30].

### 4.2. Proteins

It is well known that red seaweeds have high protein levels [34]. Reports have shown that these seaweeds have almost 47% *w*/*w* of dry matter [35]. In contrast, green algae contain moderate amounts (9–26 g protein 100 g^−1^ dry weight), while brown algae display much lower protein contents (3–15 g 100 g^−1^ dry weight) [36]. In this regard, the crude protein content of genera *Pyropia* and *Porphyra* is comparable with that of high protein plant foods such as soy [37]. It is noteworthy that the protein content of seaweeds varies not only between species [38] but also among seasonal periods [39].

The physiological effects of dietary proteins depend on the capacity of the gastrointestinal tract to digest protein intake. Digestibility of red algae proteins has been found to be moderate when compared to that of animal proteins. *In vitro* protein digestibility for *P. columbina* was studied and found to be 74.3% [40]. This value agrees with that reported by Misurcova *et al.* [41] for *Palmaria palmata* and is similar to the protein digestibility of plant proteins, but lower than that of animal proteins. This lower digestibility may be due to a high fiber content of red algae in general, and of *P. columbina* in particular (48.02% ± 1.13%), which could block the access of digestive enzymes to substrates and also decrease directly the activity of proteolytic enzymes. *In vivo* digestion has also been shown to be lower for red seaweeds. In fact, in a study in which Wistar rats were fed a diet containing similar amounts of dietary fiber (5%), protein (14%) and ash (5%), the apparent protein digestibility was lower for seaweed-fed rats and showed lower values for wakame (*Undaria pinnatifida)* than nori (*Porphyra tenera*) [8]. These data are in agreement with those found in an *in vitro* study in which the digestible, fermentable and unavailable protein fractions of these red algae were characterized and compared to that of brown seaweeds [42]. Results showed that *P. tenera* had the highest digestible protein fraction (69% *vs.* % for 28% *U. pinnatifida*), and a very small fraction of unavailable protein (3% *vs.* 17% for *U. pinnatifida*) while, when incubated with intestinal bacteria, *U. pinnatifida* fermentable fraction was higher than that of *P. tenera* (55% *vs.* 28%). Variable data were found for brown algae [42].

Although the structure and biological properties of algal proteins are still relatively poorly documented, the amino acid composition corresponding to several species of algae is known [43]. Most seaweeds contain all the essential amino acids and are a rich source of the acidic residues aspartic and glutamic acid [36]. In this regard, Cian *et al.* [40] found that the amount of aspartic + glutamic acid was 22.7 g 100 g^−1^ of proteins. Similar results were obtained for other red seaweeds such as *Pyropia acanthophora* (27 g 100 g^−1^ of proteins), *Hypnea charoides* (20.8 g 100 g^−1^ of proteins), *Palmaria palmata* (24.8 g 100 g^−1^ of proteins) and *Laurencia* species (15.5–27.4 g 100 g^−1^ of proteins) [34,44]. On the other hand, Munda [45] reported that these two amino acids can represent between 22 and 44 g 100 g^−1^ of proteins. The predominance of acidic over basic amino acids is typical of red seaweeds [34], their high levels being responsible for seaweed special flavor and taste [46].

Threonine, lysine, tryptophan, cysteine, methionine and histidine have been shown to be present at low levels in seaweeds proteins. However, research has shown that in general the concentration of these amino acids is still higher than that found in terrestrial plants [47]. Overall, the concentration of each amino acid varies to a large extent from one phylum to another, within each phylum, and even from one species to another within the same genus [43]. Amino acid levels vary with the season. Such fluctuations have been linked to a number of variables, including nutrient supply. These in turn are related to several environmental factors, such as water temperature, available light, salinity, types of proteins present and carbohydrate level [43]. Thus, Galland-Irmouli *et al.* [34] found that the glutamic acid, serine and alanine content from *P. palmata* appeared at high levels during late winter and spring, but were extremely low when harvested in July and November to January.

Lectins are a structurally diverse group of carbohydrate binding proteins of non-immune origin found in a wide range of organisms [33]. Lectins interact with specific glycan structures which are part of soluble and membrane bound glycoconjugates, and it is these protein-carbohydrate interactions that are responsible for lectin involvement in numerous biological processes such as host-pathogen interactions, cell-cell communication, induction of apoptosis, cancer metastasis and cell differentiation [48,49]. Compared to lectins characterized from animal and terrestrial plant sources, little is known about the biochemical and structural properties of *phycolectins* [33]. Of those that have been characterized, nearly all have been shown to be low molecular weight monomeric thermostable protein molecules that have affinity for oligosaccharides or glycoproteins, but not for monosaccharides, and which do not appear to require divalent cations for structural integrity or biological activity [50].

Phycobiliproteins are the main proteins of the red seaweeds, representing up to 50% of the total protein content. They are a family of fluorescent proteins covalently linked to tetrapyrrole groups, known as bilins, as prosthetic group [51]. These proteins act as antennae, absorbing energy in the visible spectrum portions where chlorophyll barely does [52]. Unlike carotenoids and chlorophylls, phycobiliproteins are not part of the photosystems located in the lipid bilayer, but constitute a structure attached to the cytoplasmic surface of thylakoid membranes named phycobilisomes.

Phycobiliproteins within phycobilisomes are weakly fluorescent. However, when they are released from the cells, they become highly fluorescent in a region of the spectrum that is well separated from the autofluorescence of other biological cell matter.

Major phycobiliproteins include phycoerythrin, phycocyanin, allophycocyanin, and phycoerythrocyanin. *R*-phycoerythrin (phycoerythrin from Rhodophyta) is an oligomeric water-soluble chromoprotein characterized by an absorption spectrum with three peaks or shoulders at 499, 545 and 565 nm [16]. It has three protein subunits: α, β and γ, whose apparent molecular weights are 18, 20 and 30–33 kDa, respectively [53]. These subunits tend to aggregate to form a basic unit that can have different arrangements. The apparent molecular weight of *R*-phycoerythrin is approximately 240 kDa. This reddish-pink pigment is of great interest because it possesses original spectral properties. Purification of this protein is performed by different techniques such as ammonium sulfate precipitation and chromatographic techniques (ion exchange, gel filtration, *etc.*). *R*-phycoerythrin is used commonly not only for applications in immunology, cell biology and flow cytometry, but also as a dye in the cosmetics industry and in natural foods. Also *R*-phycoerythrin subunits isolated from red algae can be used as a photosensitizer in photodynamic therapy of carcinoma cells [54]. The two main organisms used for the production of phycobiliproteins are the blue-green microalga *Spirulina* for phycocyanin and the unicellular red seaweed *Porphyridium* for phycoerythrin [52].

*R-*phycocyanin has a maximum absorbance at 615 nm, but also has a secondary peak at 555 nm. This peak is due to the presence of the phycoerythrobilin β subunit. The alpha subunit has phycocyanobilin as chromophore. The apparent molecular weight of R*-*phycocyanin is 110 kDa.

*R*-allophycocyanin is a phycobiliprotein found in smaller proportions in red algae. It displays an absorbance maximum at 650 nm and a shoulder at 620 nm. Its basic structure is a trimer consisting of α and β subunits (α2β) [55].

## 5. Red Seaweeds and Intestinal Mucosal Barrier Function

### 5.1. Intestinal Mucosal Barrier Function

The intestinal mucosa is the outermost layer of the four constituting the gastrointestinal tract and represents the largest body area in contact with the external environment. In addition to food digestion and absorption, serving as a mucosal barrier is one of its crucial functions. A mucus layer, consisting of a gel formed by mucin proteins and oligosaccharide chains, covers the apical surface of the intestinal mucosa. Under the mucus, the intestinal epithelium is a cell monolayer constituted by four main cell types, including columnar cells (enterocytes), goblet cells (that are the main producers of mucus), antimicrobial peptide (AMP)-producing Paneth cells and hormone producing enteroendocrine cells. In addition, the intestinal epithelium includes M cells and tuft cells. All these cell types are derived from pluripotent crypt cells located at the base of the intestinal villi. In addition, intraepithelial lymphocytes (IEL) are interspersed among the above mentioned intestinal epithelial cells. Underneath the epithelium lays the *lamina propria*, a layer of connective tissue that supports the epithelium and contains a range of immune cells including dendritic cells, macrophages and *lamina propria* lymphocytes. Finally, the *lamina propria* is surrounded by a smooth muscle layer called *muscularis mucosae*.

The central element of the barrier is the epithelium, which provides the fundamental physical limit of the mucosa, but a number of additional members contribute significantly to barrier function, including immune cells, the mucus layer, secretory immunoglobulin (Ig) A produced by plasma cells, and antimicrobial peptides. Even the intestinal microbiota can be considered a part of the mucosal barrier, since it is increasingly clear that there is a tight relationship between its presence and composition in the lumen, host immunity and overall mucosal biology. For example, the host modifies through the production of mucus, IgA or antimicrobial peptides the intestinal microbiota, while the latter is able to shape the immune response, interacting with receptors located in the intestinal epithelial cells, dendritic cells or macrophages. These receptors were initially called pathogen-recognition receptors (PPRs) and they bind pathogen-associated molecular patterns (PAMPs), *i.e.*, not specific molecules but rather types of molecules whose structure differs substantially from those in eukaryotic cells. TLRs are outstanding PPRs because of their functional relevance. Their intracellular signal transduction pathway includes the activation of nuclear factor-κB (NF-κB) and mitogen activated protein kinases (MAPKs), which result in heightened production of proinflammatory cytokines, the inhibition of apoptosis and the increase of cell proliferation [9,10].

### 5.2. Red Seaweeds and Intestinal Bacteria

Intestinal dysbiosis has increasingly been observed in a variety of intestinal and systemic diseases, and maintaining an adequate bacteria profile may be a key point to a healthy MBF [56]. Seaweed components can be used by the intestinal microbiota so as to result in prebiotic effects, potentiating the growth of beneficial bacteria in detriment of harmful microbiota [57,58,59,60]. In this regard, the administration of whole red seaweeds (*Sarcodiotheca gaudichaudii* and *Chondrus crispus*) to chicken has been described to affect the intestinal mucosa, enhancing villus height and villus surface area, as well as the intestinal microbiota, increasing the abundance of beneficial bacteria (e.g., *Bifidobacterium longum* and *Streptococcus salivarius)* and, importantly, reducing the prevalence of *Clostridium perfringens*. These microbiota modifications were accompanied by higher short chain fatty acid concentrations, pointing to an overall prebiotic effect of the seaweeds [57].

Red seaweeds have been shown to affect not only the relative abundance of bacterial species, but also bacterial translocation. *Pseudomonas aeruginosa* is an opportunistic pathogen whose translocation to the extraluminal domain is an important pathogenic phenomenon and a cause of systemic infections. A water extract from the cultivated red seaweed *C. crispus*, rich in κ-carrageenan, was administered as a food supplement to *Caenorhabditis elegans*, resulting in upregulation of the innate immune genes of the host and a decreased infection rate [61].

Both the degradation and fermentation of red seaweed oligosaccharides by the intestinal microbiota have been objects of study. In general, these compounds are poorly fermented/degraded both in humans and other mammals. As stated in the introduction, red seaweed polysaccharides contain unique structures and sulfate esters that are absent in terrestrial plants. Therefore, the intestinal bacteria are possibly not prepared or adapted to metabolize them, for instance by lacking the needed fermentative enzymes. This is well exemplified in a study in which nori and wakame, red and brown seaweeds, respectively, were given to rats, showing that their oligosaccharides are indeed degraded but poorly fermented [62]. However, it induced a bacterial adaptation that brought about a higher fermentation of these substrates over time. In spite of the above, some outstanding studies have documented the presence of red seaweed carbohydrate degrading enzymes in human gut microbiota, as well as their origin [63,64,65,66,67]. In one of these studies, the enzymes that specifically hydrolyze extracts from the agarophytic red algae *Gelidium*, *Gracilaria* and *Porphyra*, (but were inactive degrading agarose) were isolated and characterized. Notably, a search of these enzymes in the human gut metagenome indicated their presence in Japanese but not in North American individuals [65]. Of course, Japanese populations consume seaweeds in their daily diet (14.2 g per person per day), and *Porphyra* spp. (nori) is the most important nutritional seaweed, traditionally used to prepare sushi. Additional studies have further demonstrated the presence of several seaweed carbohydrate metabolizing enzymes in the microbiota of North American [63] and Spanish [64] populations. Horizontal gene transfer of enzymes to gut microbiota from marine bacteria associated to, and ingested with, these algae has been shown to be the way for these enzymes to reach the intestinal bacteria [65,66].

To discern whether polysaccharides from red seaweeds are degraded or not is important not only for their prebiotic effect but also because many of their functional properties have been shown to depend on the molecular weight of the colloid and the amount of sulfations along the polysaccharide chain. This is illustrated by a study in which the hydrolysis of porphyran alpha-1,3 linkages brought about free radical scavenging capacity, whereas the hydrolysis of beta-1,4 linkages did not increase the antioxidant activity markedly [68]. The importance of sulfations is exemplified in a study in which a polysaccharide acid soluble fraction of *Porphyria yezoensis* was obtained and the activation of macrophages was tested. Desulfation of this fraction decreased macrophage activation, while the digestion with β-agarase increased it [69].

Although not directly related to intestinal bacteria or with intestinal epithelial barrier function, it is worthy to comment on some articles in which antiviral effects of red algae ι-carrageenan were described [70,71,72]. These articles are valuable because they were carried out in humans and because common cold producing viruses alter airway MBF as some viruses do with the intestinal MBF. In these double blind, randomised, placebo-controlled studies, ι-carrageenan was administered to either adults [70,71] or children [72] with acute symptoms of the common cold. The results indicate that the administration of the carrageenan diminished the viral load in nasal secretions. Furthermore, in adults it reduced the duration of cold symptoms [70,71] and the relapses [70], while in children the incidence of secondary infections was reduced [72]. In a similar study with 35 adults suffering from early symptoms of common cold, ι-carrageenan showed comparable effects, diminishing viral load in nasal secretions, and also reducing the mucosal levels of the pro-inflammatory mediators fibroblast growth factor (FGF)-2, fractalkine, chemokine (C-X-C motif) ligand 1 (CXCL-1 or GROα), granulocyte colony-stimulating factor (G-CSF), interleukin (IL)-8, IL-1α, interferon (IFN) γ-induced protein 10 (IP-10), IL-10, and IFN-α2 [73]. Other antiviral effects of carrageenans have been described and reviewed elsewhere [74].

### 5.3. Red Seaweeds and the Intestinal Epithelium

The equilibrium among epithelial cell proliferation, differentiation and death is an important feature of the intestinal MBF [21]. In pathologic processes like inflammatory bowel disease apoptosis of intestinal epithelial cells is markedly increased [75]. Challenges to the epithelial monolayer by microorganisms, inflammation, toxic luminal substances, and so forth, impose the need for appropriate mechanism to preserve MBF despite the occurrence of gaps [10].

Food derived bioactive peptides have been shown to modulate intestinal epithelial cell differentiation and cytokine production [76]. Among these, a bioactive peptide derived from *Porphyra yezoensis*, termed PY-PE or PYP1, whose sequence is A-L-E-G-G-K-S-S-G-G-G-E-A-T-R-D-P-E-P-T, has been shown to induce the proliferation of IEC-6 cells, a rat intestinal epithelial cell line [77,78]. The stimulation of two different molecular signaling pathways has been found to be involved in this effect. Epidermal growth factor (EGF) is an important factor in the regulation of MBF, as it stimulates epithelial cell growth, proliferation and differentiation by binding to its receptor (EGFR) and the subsequent activation of several signaling pathways. PY-PE has been shown to increase the expression of EGFR and the adaptor molecules Shc, growth factor receptor bound 2 (Grb2) and Son of Sevenless (SOS), and to activate the Ras/Raf/mitogen activated protein kinase (MAPK)-ERK kinase (MEK)/extracellular signal-regulated kinases (ERK) pathway (Ras/Raf/MEK/ERK) [77]. The final effect is the recruitment of cells into G1 phase, with an increased expression of cyclins D and E, cyclin dependent kinase (CDK) 2, 4 and 6, and a decrease in the expression of cell cycle inhibitors p21 and p27. Peptide PY-PE also stimulates the expression of type I IGFR, which is involved in the promotion of cell proliferation and the inhibition of apoptosis [77], and of the adaptor molecules insulin receptor substrate-1 (IRS-1) and Shc. As a result PY-PE stimulates the phophatidylinositol 3-kinase (PI3K)/protein kinase B (PKB or AKT) pathway, ERK, and the expression of AP-1 proteins c-Jun and c-Fos [78]. Therefore PY-PE induces cell proliferation through the stimulation of different receptor and signal transduction pathways in IEC-6 cells [77,78].

Other peptides and proteins derived from *P. yezoensis* have been shown to have effects on cell proliferation and apoptosis that vary depending on the cell/animal used for the study and the experimental conditions. For example, peptide PPY, whose sequence is KKAAE, induces cell cycle arrest and activates apoptosis in a mammalian cancer cell line (MCF-2), decreasing the expression level of type I IGFR in a concentration dependent manner [79]. On the other hand, a 14 kDa protein isolated from this red alga has been shown to reduce caspase-3 activity in rats with acetaminophen induced liver injury [80].

Although native carrageenans are thought to be harmless (see above). *In vitro*, non-degraded carrageenans have been shown to exert inhibitory effects on epithelial proliferation at very low concentrations (1–10 mg/mL, lower than luminal concentrations expected from a Western diet). These effects included the induction of cell death and cell cycle arrest in the intestinal epithelial cell line NCM460 [81]. However, some studies have shown that carrageenans can produce inflammatory effects. In general, it is important to point out that controversial reports may be the consequence of studies carried out with commercially available products that contain mixtures of carrageenan of various sources and types, and of various degrees of purity and/or fractionation [61,82].

Degradation as a result of *ex vivo* treatments or of exposure to gastric and intestinal digestion or gut bacteria degradation after ingestion may produce in turn harmful forms of carrageenan. In fact, acid treatment at high temperature (80 °C) triggers carrageenan hydrolysis to lower molecular weight (<50 kDa) compounds known as poligeenan or degraded carrageenans. These degraded carrageenans induce inflammation and have been actually used as models of colitis in several species, including rats [83,84], rabbits [85] and guinea pigs [86]. In fact, the Scientific Committee on Food of the European Commission advised and the European Commission adopted the recommendation that the content of carrageenan of size less than 50,000 Da in food products should not exceed 5%, if feasible [87]. Not all degraded carrageenans are harmful, however. For example, a 10 kDa carrageenan fraction obtained from native ι-carrageenan from *Euchema spinosum* by acid hydrolysis at high temperature did not induce colitis in rats while a 50 kDa fraction was colitogenic [84]. Inhibition of cell proliferation and induction of apoptosis was shown by a hydrocholic acid degraded κ-carrageenan in intestinal epithelial (Caco-2 and FHs 74 Int) and liver (HepG2 and Fa2N-4) cell lines [88].

*In vitro* experiments have shown that carrageenans (native and degraded) may also affect epithelial cell immune response. Thus commercial carrageenans (λ, ι and κ) induce an increase in IL-8 production in intestinal epithelial cells [89,90]. A further study of the lambda form revealed a stimulation pathway that involved the activation of the BCL (B-cell lymphoma/leukemia)10-NF-κB signaling pathway, not only in the human normal colonic mucosal epithelial cell line (NCM460), but also in *ex vivo* human colonic tissue, and in primary human colonic epithelial cells [89]. Similar results have been obtained for enzyme degraded carrageenans, although the effects depend on the particular type of degradation [91]. This *in vitro* study with the NCM460 cell line showed that hydrolysis of carrageenan with the enzyme α-1→(3,6)-galactosidase significantly reduced the increase in IL-8 and BCL10, while specific κ- or ι-carrageenases, which hydrolyze the β-1,4-galactosidic bonds, induce IL-8 and BCL10 production, presumably because they increase the exposure of the immunogenic α-1→3-galactosidic epitope of carrageenan to Toll like receptor (TLR) 4, with the subsequent stimulation of NF-κB [91]. Together, the above results indicate a pathway that involves TLR4/BCL10/NF-κB and results in modulation of IL-8 production in intestinal epithelial cells. The participation of this signal transduction pathway in the induction of proinflammatory cytokines by carrageenans has been confirmed in other studies with intestinal epithelial cells and macrophages (see below) [90,92].

*In vitro* studies have shown that the contact of κ-carrageenan with macrophages stimulates the immune response and induces epithelial cell pro-inflammatory cytokine production, apoptosis, and the disruption of the intestinal epithelium. In fact, in an interesting *in vitro* experiment with cocultures of macrophages (THP-1 cells) and Caco-2 cells (an intestinal epithelial cell line), treatment with κ-carrageenan increased the secretion of tumor necrosis factor (TNF)-α, IL-1β, and IL-6 and resulted in apoptosis and reduced transepithelial electrical resistance of the epithelial layer [93]. When the TNF-α receptor 1 was blocked with antibodies, changes in IL-1β and IL-6 levels, apoptosis and transepithelial electrical resistance were attenuated, indicating that macrophage produced TNF-α in response to κ-carrageenan was contributing to Caco-2 monolayer damage.

In general, an increase in proinflammatory cytokine production is viewed as a factor tilting the balance toward inflammatory state. Nevertheless, growing evidence suggests that inducing proinflammatory cytokine production in intestinal epithelial cells, secondary to the activation of TLR and NF-κB, may be of physiological importance to maintain an adequate state of basal immune activation and epithelial proliferation that allows the intestine to be protected. An important piece of evidence that supports this theory is the fact that the absence of TLRs or their related transducing proteins in knock-out mice results in a spontaneous colitis or in a higher susceptibility to it [9,10]. Furthermore, increased IL-1β, IL-6 and TNF-α production appears to have protective effects in experimental colitis induced by the administration of dextran sulfate sodium, which has been attributed to a proliferative effect on epithelial intestinal cells [94,95,96].

In general, cancerous cells and adenomatous polyp cells, the latter recognized as potential precursors of colorectal cancer, have characteristic features including increased proliferative activity with concomitant reduced differentiation phenotype and reduced apoptotic ability [97]. Interestingly, red seaweed algae multimineral extracts from different *Lithothamnion* species have been shown to inhibit polyp formation in animal models (see below). Although the mechanism of action is not well defined, *in vitro* studies indicate that an extract from *Phymatolithon calcareum* (formerly *L.calcareum*) inhibits Ki67 antigen (a proliferation marker) and promotes differentiation (increased E-cadherin staining) in human colon tissue in organ culture [98]. Similar antiproliferative and pro-differentiation effects were observed in human colon carcinoma cell lines when cultured with an extract that contained 12% Ca^2+^, 1% Mg^2+^, and detectable amounts of 72 trace elements [99]. Although calcium may be in part responsible for the observed effects, some of the trace elements (lanthanides in particular) have been proposed to enhance the growth control properties of calcium. In this sense, it has been shown that gadolinium increases the growth inhibitory properties of calcium on intestinal epithelial cells, without affecting calcium-induced differentiation [100]. Mechanisms involved in this effect remain to be elucidated.

### 5.4. Red Seaweeds, Oxidative Stress and Macrophage Stimulation

The effects of red seaweeds on macrophage activation depend on the alga species and on the composition of the preparation studied. In general red seaweed proteins, peptides and polysaccharides exert antiinflammatory and antioxidant effects on macrophages.

*Pyropia columbina* (formerly *Porphyra columbina*) has been studied as a source of protein and bioactive peptides [54,101]. A protein hydrolysate from this seaweed that resulted from the sequential digestion of an extract with fungal proteases and flavourzyme was found to induce IL-10 expression (an anti-inflammatory cytokine) in splenocyte preparations, which mainly contain splenic antigen presenting cells (with a high percentage of macrophages) and lymphocytes. Inhibition of proinflammatory cytokines (TNF-α when stimulated with LPS and IFN-γ in basal conditions and under concanavalin A (ConA) stimulation) was also observed in these cells. When macrophages and lymphocytes were isolated from the spleen and studied separately there was again an increased production of IL-10, both in basal conditions and after the activation of macrophages with LPS and of lymphocytes with ConA [101]. A decrease in proinflammatory cytokine production (IL-6 and TNF-α) in LPS stimulated macrophages was also observed, while IFN-γ secretion was diminished in isolated lymphocytes both under basal and stimulated conditions. IL-10 is an anti-inflammatory cytokine mainly produced by monocytes/macrophages and to a lesser extent by lymphocytes (Th2 and T regulatory) and mastocytes. The effects of IL-10 include the inhibition of lymphocyte differentiation to Th1 cells, which produce IFN-γ, which is consistent with the observed results. The effects of the *P. columbina* hydrolysate were mediated by MAPK p38 and NF-κB, which are involved in IL-10 induction [102].

Notably, full protein fractions of *P. columbina* have been shown to exert similar effects, namely induction of rat splenocyte proliferation and IL-10 secretion, observed also in isolated macrophages and specially T lymphocytes [54]. Furthermore, these effects were not attributable to either R-phycoerytrhrin or C-phycocyanin, phycobiliproteins from red seaweeds that are known to have immunomodulatory effects, since both markedly diminished IL-10 production. Again in this study the involvement of JNK/p38 MAPK and NF-κB dependent pathways in macrophages and lymphocytes was established.

Other red seaweed proteins may exert antiinflammatory effects on macrophages. PGP, a glycoprotein isolated from *P. yezoensis*, has been shown to inhibit proinflammatory cytokine (TNF-α and IL-1β) production in LPS stimulated RAW 264.7 mouse macrophages. This effect was accompanied by the inhibition of NO and ROS production and of the expression of iNOS and COX2. Mechanistic studies showed that, in accordance with the studies with *P. columbina* peptides and proteins, this glycoprotein inhibited the stimulation of TLR4, NF-κB and MAPK (ERK1/2 and JNK) signal transduction pathways by LPS to produce these effects [103].

Inhibition of macrophage proinflammatory cytokine production was also induced by a methanolic extract of *Gracilaria changii* that contained chlorophyll proteins, methyl 10-hydroxyphaeophorbide and 10-hydroxypheophytin, and several other unidentified molecules. This extract significantly reduced the expression of TNF-α and IL-6 in U937 cells treated with *phorbol myristate acetate (*PMA). Of note, no cytotoxic effects were recorded for cells treated with the 10 μg/mL of this extract [104]. Interestingly, a protective effect was also observed *in vivo*, reducing absolute ethanol-induced gastric lesion sizes by >99%, when fed to rats. This effect was associated to increased stomach pH and augmented non protein sulfhydryl (NP-SH) levels. A lowered gastric pH has been associated both to ethanol-induced acute gastric mucosal injury and to alterations in the intestinal microbiota potentially leading to diarrhea and oxidative damage. In fact, the amount of NP-SH (together with antioxidants like reduced glutathione) is decreased in models of gastric injury [105]. Of note, *G. changii* has been reported to have free radical scavenging properties [106,107].

Porphyran has antioxidant activities, as demonstrated by its free-radical and anti-inflammatory activities. *P. yezoensis* is a good source of porphyran with high scavenging activity toward superoxide anion and hydroxyl radical. Some studies have shown that *P. yezoensis* porphyran inhibits the expression of nitric oxide synthase. However, the effect on the production of nitric oxide varies depending on the porphyran studied [108,109]. In two studies from the group of Oda, porphyran from normal nori (the sheeted food stuff used in sushi) and porphyran prepared from a discolored waste nori (dc-porphyran) were used. The latter is characterized by having a greatly reduced molecular mass. While both types inhibit the expression of nitric oxide synthase, only dc-porphyran inhibits the consequent production of nitric oxide in LPS-stimulated RAW264.7 cells [88]. Likewise, dc-porphyran showed a slightly higher antioxidant activity. These results are in accordance with the idea that the molecular size is important for the effects of phycocolloids in general, and porphyran in particular. Different strategies have been successfully used to decrease the molecular weight of porphyran with the final goal of increasing the antioxidant activity, including ultrasonic treatment [110] or enzymatic hydrolysis [68]. The latter has shown that when alpha-1,3 linkages are hydrolyzed the antioxidant activity with regard to free-radical-scavenging capacity and, specifically, superoxide radical anion scavenging activity, is increased, whereas the hydrolysis of beta-1,4 linkages has little effect [68].

In contrast to the inhibitory effects of proteins and peptides on macrophage activity, porphyran enriched fractions from *P. yezoensis* induce macrophage activation [69,88]. As we indicated above, the sulfations are important in the effect of red seaweed oligosaccharides on the activation of macrophages. In this regard, an acid-soluble polysaccharide fraction of *P. yezoensis* was obtained and the activation of macrophages was tested. Desulfation of this fraction decreased macrophage activation while the digestion with β-agarase increased their activation [69].

### 5.5. In Vivo Effects of Red Algae Derived Products

Sulfated polysaccharide fractions from different *Gracilaria* species have been shown to be beneficial in animal models characterized by direct alterations in MBF and inflammation. These include the model of colitis induced by the administration of trinitrobenzenesulfonic (TNBS) acid to rats [111] and models of damage induced by naproxene [112] or ethanol [113]. The inhibition of inflammatory cell infiltration, cytokine release and lipid peroxidation have been proposed as molecular mechanisms of action [104].

Related to antiallergic activity, porphyran has been found to be effective against different allergic responses. According to Ishihara *et al.* [114], oral administration of porphyran (2% in drinking water) from the red algae *Pyropia tenera* (formerly *Porphyra tenera*) and *P. yezoensis* are capable to inhibit the contact hypersensitivity reaction induced by 2,4,6-trinitrochlorobenzene, decreasing the serum level of IgE in BALB/c mice.

*Lithothamnion muelleri* is a red alga that, when fed to mice, has been shown to have antiinflammatory effects in several models of disease, including arthritis [115] and graft *versus* host disease [116]. Mineral extracts from other species of *Lithothamnion* have been found to ameliorate the spontaneous development of colitis and the severity of disease in IL-10 knockout mice on a C57BL/6J background [117] and to reduce colonic inflammation and polyp formation in the gastrointestinal tract of C57BL/6 mice fed a high-fat diet [118,119]. Finally, as commented above, the methanolic extract of *G. changii* has gastroprotective properties in rats, an action attributed to antioxidant mechanisms [106,107].

## 6. Functional Foods Incorporating Red Seaweeds

To date, there is no standard definition for functional foods. According to the Food and Agriculture Organization [120], functional foods are those foods similar to conventional foods in appearance, intended to be consumed as part of a normal diet containing biologically active compounds which offer potential for enhanced health or reduced risk of disease. For nutraceutical and/or dietary supplements, no consensual definition is found either and there is still ambiguity about the regulatory requirements related to nutraceuticals [121]. Nevertheless, a common aspect is that in all of these products the main focus is on improving health and reducing disease risk through prevention towards improvement of quality of life and well-being contributing to an increased health and longevity [122]. Nowadays, consumers are increasingly aware of the relationship between diet, health and disease prevention.

The terrestrial environment (e.g., fruits, vegetables, cereals and mushrooms) as a reservoir of bioactive compounds is by far more explored than the marine counterpart (e.g., fish, sponges, macro- and microalgae). Although many functional marine ingredients are presently known, it is believed that multiple other marine ingredients remain to be evaluated and new sources to be discovered. Thus, the marine environment is a major reservoir of bioactive compounds that have the potential to be applied in several phases of food processing, storage and fortification [123]. The variable characteristics of marine environments such as degree of salinity, temperature, pressure and illumination, impart particular interest on compounds derived from marine organisms.

Red seaweeds are a very interesting natural source of compounds with biological activity that may be used as functional ingredients, and considering their great taxonomic diversity, research on the identification of biologically active compounds from algae can be seen as an almost unlimited source. Moreover, such extracts are virtually fat and calorie-free, making them increasingly sought for commercial purposes. For instance, macroalgae, e.g., *Pyropia ternera*, have been found to be good sources of dietary fiber associated with changes in microbial activity that involve a decrease in reductive and hydrolytic enzymatic activities implicated in the conversion of procarcinogens into carcinogens in rats. In this regard, the combination of the effect on the gut flora and a more rapid transit of feces would be expected to reduce exposure to potential carcinogens and may have health implications in human nutrition [62]. On the other hand, carrageenans and agar have been known to act as modulators of coagulation as well as to display antithrombotic, anti-inflammatory, antioxidant, anticancer and antidiabetic activities, among others. These soluble polysaccharides from red algae have tremendous potential as dietary fiber for human nutrition and are being evaluated as new possible prebiotic compounds [124].

In addition to their potential use as fiber/prebiotics, enhancement of antioxidant activity and immunity stimulation are the most studied health benefits and have driven consumers to be more aware that diet can serve both nutrition and health promoting goals. Food products containing marine derived oils rich in omega-3 fatty acids, chitin, chitosan, *etc.* are some among the ones commercialized in several markets around the world including the United States, Japan and some countries in Europe [125].

Besides the scientific interest in the use of algae functional ingredients, there are various challenges ahead that have to be overcome to use them in new functional foods. Foods should have good sensorial characteristics in order to be accepted by the consumers since very few of them are willing to compromise taste for healthiness in food [126]. To our knowledge, there is still a lack of research in the application of such functional/bioactive ingredients in foods as well as the scientific validation of their technological and biological feasibility. The design of functional foods based on the incorporation of algae ingredients has been more successful in bakery, pasta and extruded maize products. In this regard, Prabhasankar *et al.* [127] found that the addition of edible seaweed wakame (*U. pinnatifida*) up to 20% had sensorial acceptance and resulted in improved amino acid and fatty acid profiles, increased antioxidant activity, and a higher content of fucoxanthin and fucosterol in seaweed pasta [127]. Cian *et al.* [128] developed an extruded maize product added with a red seaweed *Pyropia columbina* (3.5%) and evaluated *in vitro* the presence and resistance to gastric digestion of bioactive compounds (angiotensin-converting enzyme inhibitors and antioxidants). This study suggests that the bioavailability of bioactive compounds is enhanced in snacks added with algae. Additionally, these authors studied the effect of the extruded maize product added with the red seaweed *P. columbina* on colonic health, lipid metabolism and oxidative status in growing Wistar rats. They found that addition of red seaweed had antioxidative effects on the liver, reducing thiobarbituric acid reactive substances (TBARS) and oxidized glutathione and increasing the redox index and catalase levels. Also, the researchers found a reduction in bacterial mucinase activity and in cyclooxigenase-2 (COX-2) and inducible nitric oxide synthase (iNOS) expression, and an increase in cecal IgA levels.

It is noteworthy that the introduction of seaweeds in the human diet will always be a complex subject due to several types of constraints, such as diet type and habits, which are related to cultural and ethnic aspects of the population, consumer ideas and fears about sea pollution, and also legislation itself. For example there are different views with respect to marine sources as functional foods; edible seaweeds are a product with a very long tradition in human diet in Japan, China and Korea, and also in the USA as a consequence of the East to West migration phenomenon, whereas in Europe, although France has placed great effort in getting these products approved for human consumption, some countries still present legal obstacles that may delay approval [47]. Taking all this into account, consumer acceptance of new functional foods with seaweeds will certainly be dependent on the balance between habits and traditions, their perception about the real health benefits of functional foods and, as previously mentioned, organoleptic issues. Additionally, the commercialization of bioactive compounds or functional foods with health claims implies an extensive scientific dossier providing sufficient scientific evidence, which is highly expensive and burdensome, and the niche market targeted may not be large enough to cover the economical investment [129].

## 7. Conclusions

Based on their composition, marine algae, and specifically red seaweeds, are good potential functional foods. Among their unique components, proteins and derived peptides together with polysaccharides and minerals have the ability to balance the MBF, acting as prebiotics, regulating intestinal epithelial cell, macrophage and lymphocyte proliferation and differentiation, and modulating the immune response. Although molecular mechanisms of action are starting to be elucidated, evidence indicates the involvement EGFR, IGFR, TLRs and signal transduction pathways mediated by AKT, NF-κB, and MAPKs. The need for further research is clear, but *in vivo* experiments point to an overall antiinflammatory effect of these algae, indicating that they can reinforce the MBF.
